# Finger flexor rigidity in the healthy population

**DOI:** 10.1038/s41598-024-52971-y

**Published:** 2024-02-05

**Authors:** Erez Grinbaum, Igor Shulman, Nimrod Rozen, Guy Rubin

**Affiliations:** 1 Orthopedic Department, Emek Medical Center, Afula, Israel; 2 Faculty of Medicine, Technion, Haifa, Israel

**Keywords:** Tendons, Preclinical research

## Abstract

The involvement of the hand flexors in trigger finger is not clear. This study aimed to examine the rigidity of the flexor tendon in the first pulley territory in the hand by using ultrasound in a healthy population, as well as to create a reference scale of rigidity for the flexor tendons to compare those values in trigger fingers. We tested 35 healthy volunteers using a linear ultrasound transducer and the color Doppler method. Rigidity levels below the first pulley were examined and compared between the different fingers of the hand and the relationship between rigidity and sex and the three different age groups was evaluated. In the healthy population, the rigidity of the flexor tendons of the hand in the territory of the first pulley varied between 233.1 and 962.8 kPa, with an average of 486.42 kPa and standard deviation of 114.85. We showed that the flexors in the dominant hand were more rigid, there was a difference between the rigidity of the flexor tendons of the thumb and the other fingers of the same hand, and the ring finger of the dominant hand had stiffer flexor tendons than the fingers of the other hand in the male population. We created a value scale for the rigidity of the flexor tendons of the fingers. This base scale can be compared between different pathologies, including trigger finger. The study and all experimental protocols were approved by the local ethical committee.

## Introduction

Trigger finger is one of the most common pathologies in hand surgery. The entrance to the flexor tunnel in the palm is called the A1 Pulley. Trigger finger is a phenomenon in which the entrance to the flexor pulley in the hand is thickened and tendon movements are restricted, causing pain and locking of the finger upon bending. This condition can occur in a single finger or simultaneously in several fingers. The prevalence of trigger finger in the diabetic population is estimated to be approximately 17% in patients with diabetes, while in the non-diabetic population it is estimated to be approximately 3% in the population aged over 30 years^1^. Additionally, the prevalence is six times higher in women than in men. The distribution of trigger finger between the fingers is 50% for the thumb, 7% for the second finger, 28% for the middle finger, 14% for the fourth finger, and 2% for the fifth finger^2^. Secondary trigger finger is common in patients with diabetes, gout, kidney disease, and arthritis, and is related to a worse prognosis in conservative and surgical management^3–5^.

In recent years, the first pulley (A1) has been characterized by ultrasound, using sensitive transducers^6^. Classic ultrasound findings of trigger finger include the nodular form or global hypoechogenic thickening of the A1 pulley, hyperemia of the pulley on Doppler, and cystic changes of the pulley in advanced cases^7^. Moreover, a previous study found that in the trigger finger, the A1 pulley was significantly thickened compared to that in the control group^8^. In addition, it has been demonstrated that the flexor tendon is significantly thickened in trigger finger when compared to a finger with no pathology, and there is a thicker tendon in those with limited straightening of the finger than those with no limitation^9^. Ultrasound is also used to evaluate the rigidity of soft tissue using the sonoelastography (SEG) method^10,11^. This technique can assess the level of rigidity in the musculoskeletal area, including fibrotic tissue. Two SEG techniques are used to evaluate tissue rigidity. The first is the static method, which involves pressing and creating a strain that is translated to the Young’s modulus of elasticity curve, and the second uses the progression of the vibration speed and its translation to the Young’s modulus^11^. Using the SEG technique, previous studies have demonstrated that tendon rigidity is increased in patients with Achilles tendinitis and lateral epicondylitis^12,13^.

Recently, the SEG technique was used to demonstrate the level of rigidity of pulley A1 (the ratio between the strain in the tissue above the pulley and pulley tissue), and it was shown that the level of rigidity increased with age and the presence of trigger finger. Moreover, it was shown that 3 weeks after steroid injection, the finger was no longer stuck, and the pulley rigidity decreased^14^.

Furthermore, hypoechogenic findings in the superficial flexor tendon were recently demonstrated on ultrasound in advanced cases of trigger finger that usually include contracture of the proximal interphalangeal joint^7^. These findings support our hypothesis that there is a pathology of the flexor tendon, in addition to the pulley, in advanced cases of trigger finger, which may be demonstrated as an increase in flexor rigidity.

To our knowledge, there is currently no existing reference scale for the rigidity of the digital flexors of the hand in the first pulley territory in a healthy population. Therefore, this study aims to create a baseline scale for a healthy population using a directed ultrasound test. This may allow the future comparison of different pathologies, such as trigger finger, to this scale.

## Materials and methods

The study was performed in accordance with the Declaration of Helsinki ethical approval ID 0062-20-EMC. Volunteers were recruited, and the inclusion criteria included healthy adults aged 18–80 years without diabetes, arthritis, trigger finger, or prior hand surgery. The exclusion criteria encompassed volunteers under the age of 18, patients who had previously undergone hand and finger surgery, as well as those with rheumatic diseases, storage diseases, gout, and diabetes. Volunteers who met the relevant criteria signed an informed consent form to participate in the study.

Thirty-five volunteers were recruited and completed the personal questionnaire, which included pre-existing conditions, occupation, and trigger finger-directed anamnesis, and underwent physical examination by an orthopedic surgeon. The ultrasound test was performed by a radiologist while the participant was seated on a chair with their hand on a bed, elbow bent at a 90-degree angle, and the palm fixed in a designated apparatus with a straight finger.

**Figure 1:**
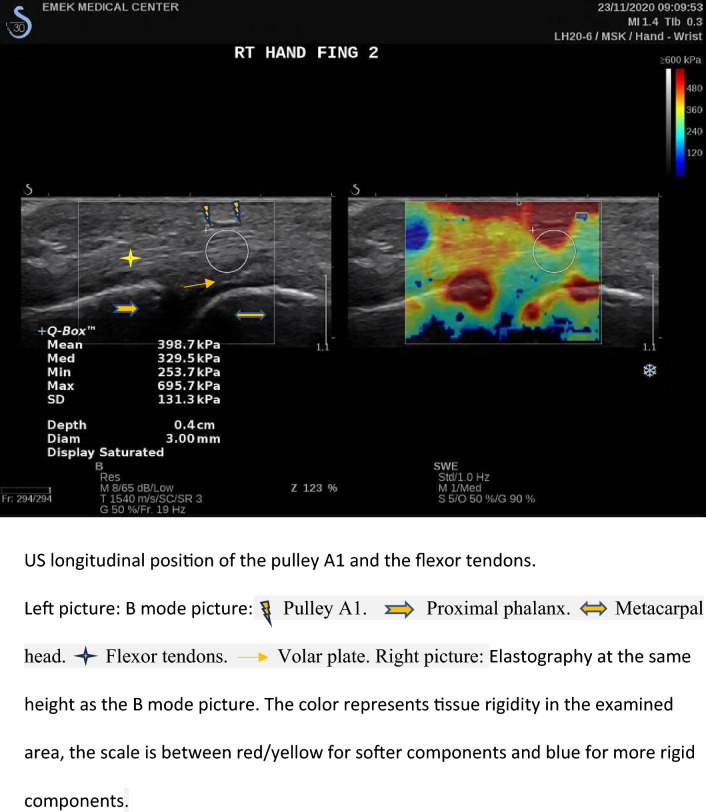
The flexor tendon in a longitudinal section below the pulley A1 in an ultrasound examination and elastography (measuring rigidity).

The fingers were examined using a hockey stick linear transducer 6–20 MHz, in gray scale sonoelastography, as well as the color Doppler method (Supersonic Imagine SA: Provence, France)^10,11^. After applying a generous amount of gel on the tested finger, the transducer was placed longitudinally on the palmar aspect of the hand, and tendon rigidity was measured below the pulley surface, with elastography in kPa units (Fig. 1). The measurements were always performed in the same order and the results were recorded.

After gathering the data, we compared the measurements between all fingers and searched for statistically significant differences. Subsequently, we tested whether there were significant differences according to sex and age groups (aged 19–39, 40–59, and 60–80 years).

### Statistical analysis

Based on a pilot study, a paired samples t-test was utilized to estimate the required sample size. For a desired power of 0.80 and a Type I error rate of 0.05, the analysis indicated a need for 128 fingers (64 from each hand). This sample size would enable the detection of a mean difference of 10, considering a standard deviation of the difference of 40 in the degree of stiffness of the flexor tendon between the fingers of the right hand and the corresponding fingers of the left hand. The sample size analysis was conducted using Gpower 3.1. This study included 35 participants, with a statistical test power of at least 80%. In order to assess whether there was a difference in the average flexor tendon rigidity between the two palms, we created two variables and used a paired t-test.

Moreover, to investigate whether there was a difference in tendon rigidity between the fingers of the same hand, we used repeated measures of post hoc Bonferroni tests.

We performed paired t-tests to compare the dependent variables between the fingers of the right and left hands of the participants. The results were considered statistically significant when the p value (paired t-test) and adjusted p value were less than 0.05. All tests were two-tailed.

### Ethics approval and consent to participate

The study and all experimental protocols were approved by the “Emek” medical center ethical committee. All methods were carried out in accordance with relevant guidelines and regulations. Written informed consent was obtained from all participants.

## Results

Between the years 2020 and 2022, 35 healthy volunteers who met the inclusion criteria were recruited. The average age of the participants was 43.6 years (19–77), with 17 women (48.6%) and 18 men (51.4%). Thirty-three participants (94.2%) were right-handed, and only two participants (5.7%) were left-handed. The participants were divided into three age groups: the first, aged 19–39 years, included 18 participants (51.4%); the second, aged 40–59 years, included 10 participants (28.5%); and the third, aged 60–80 years, included 7 patients (20%). The average tendons rigidity upon ultrasound ranged between 233.1 and 962.8 kPa, with a standard deviation of ± 114.85 kPa (Table 1).

**Table 1:** Characterization of the participants in the study and the mean flexor tendon stiffness and standard deviation.

N	35
Age distribution	
Average age	43.6 (14.97)
Age 19–39	18 (51.4%)
Age 40–59	10 (28.5%)
Age 60–80	7 (20%)
Stiffness (kPs)	233.1–962.8
Mean (SD)	486.41 (114.85)
Range	233.1–962.8
Gender	
F	17 (48.6%)
Age 19–39	9 (52.9%)
Age 40–59	6 (35.2%)
Age 60–80	2 (11.7%)
M	18 (51.4%)
Age 19–39	9 (50%)
Age 40–59	4 (22.2%)
Age 60–80	5 (27.7%)
Right dominance	33 (94.2%)
Left dominance	2 (5.71%)

When we examined the difference in flexor tendon rigidity between the two palms, we found a significantly increased average flexor tendon rigidity in the right hand than that in the left hand [t (34) = 2.26; p > 0.03; confidence interval: 3.29, 61.42; mean = 32.36).

**Figure 2:**
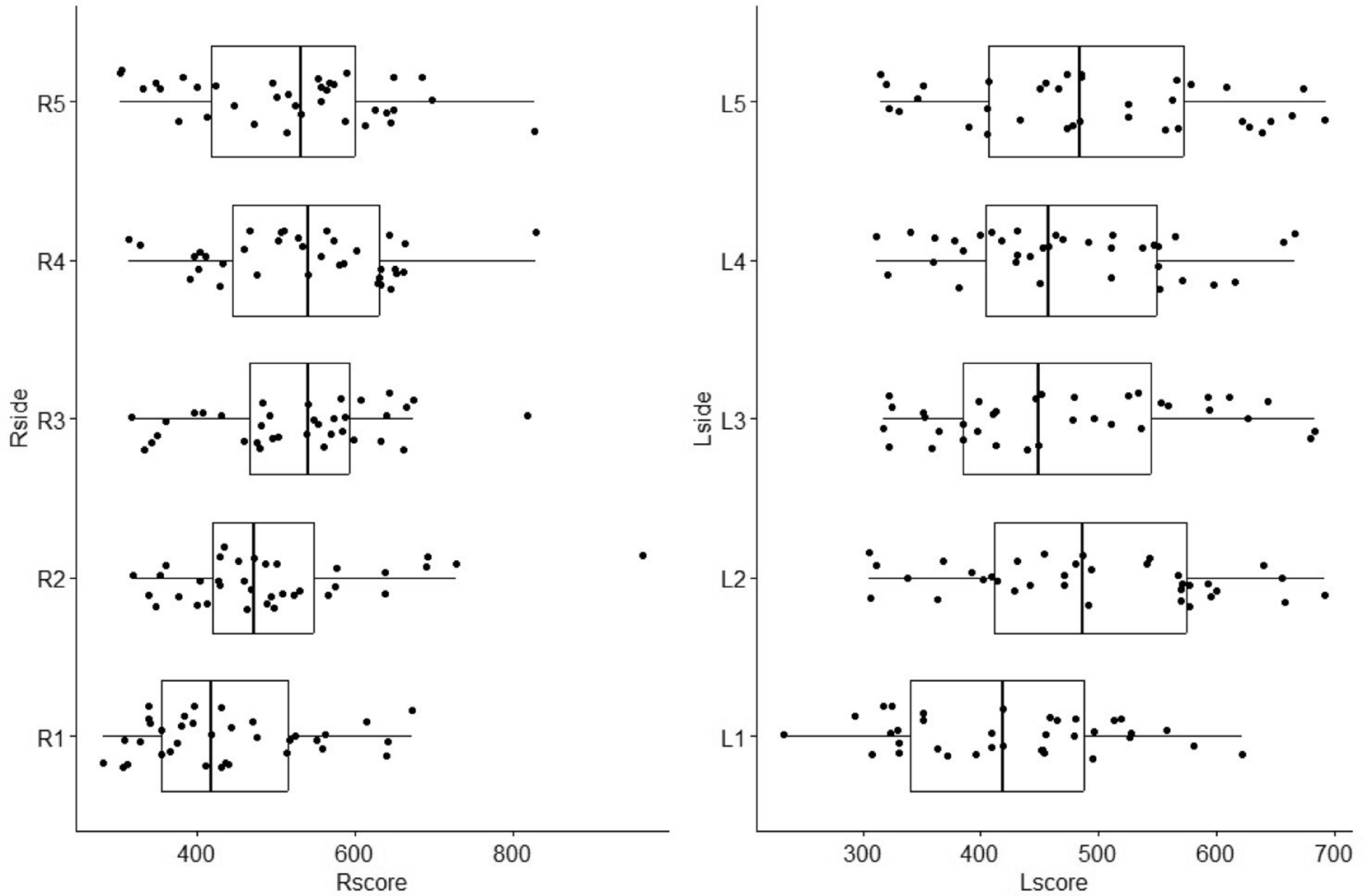
Comparison of the average rigidity of the flexor tendons of between the two hands.

We observed variations in flexor rigidity in the right hand between the thumb and middle (p = 0.026) and ring fingers (p = 0.002). In the left hand, differences were noted between the thumb and index (p = 0.04) and fifth fingers (p = 0.02) (Fig. 2).

**Figure 3:**
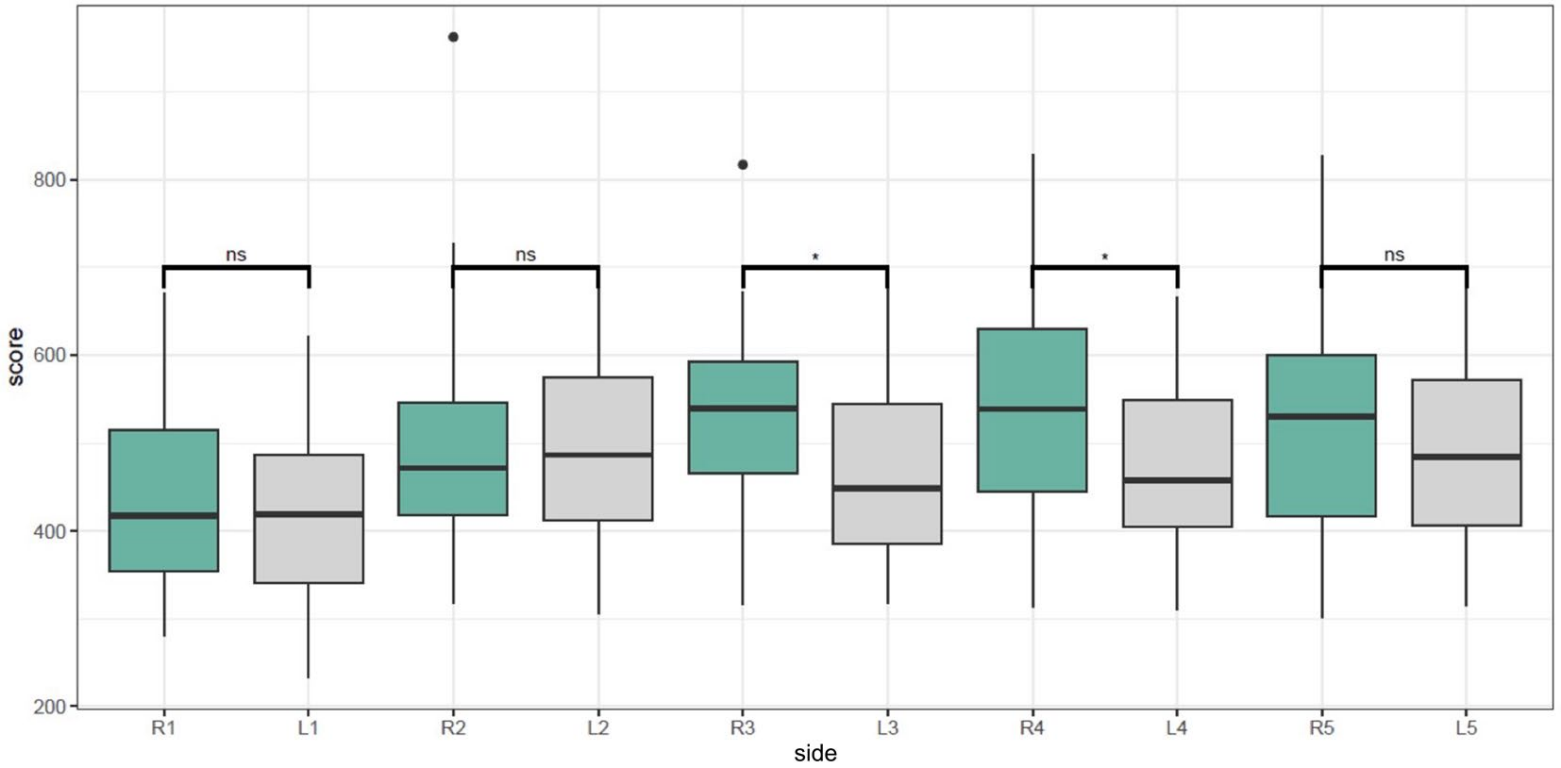
Comparison of the average rigidity of the flexor tendons between the fingers of the hands.

In comparing the flexor tendon rigidity of the left and right thumbs, no significant difference was found [t (34) < 0.57]. In addition, the flexor tendon in the right thumb was distinctly different from the flexor tendons in each finger of the right hand (Table 2). Similarly, it was found that the tendon rigidity in the left thumb was distinctly different from that in the other fingers of the left hand (Fig. 3, Table 2). No significant gender-based difference was found between the thumbs and other fingers.

**Table 2:** Comparison between flexor tendon of the thumbs and the flexor tendons in each finger of the same hand.

Pairs (R = Right hand, L = Left hand, # = Finger number)	Mean difference (CI 95%)	t-test	p value
R1–R2	− 61.04 (− 122.48, 0.4)	− 2.019	0.051
R1–R3	− 87.78 (− 142.77, − 32.79)	− 3.244	0.003
R1–R4	− 98.63 (− 147.53, − 49.73)	− 4.099	0.000
R1–R5	− 83.10 (− 144.44, − 21.76)	− 2.753	0.009
L1–L2	− 68.56 (− 114.69, − 22.44)	− 3.021	0.005
L1–L3	− 45.06 (− 85.92, − 5.11)	− 2.290	0.028
L1–L4	− 49.06 (− 93.79, − 4.33)	− 2.229	0.033
L1–L5	− 72.05 (− 117.54, − 26.57)	− 3.219	0.003

**Figure 4:**
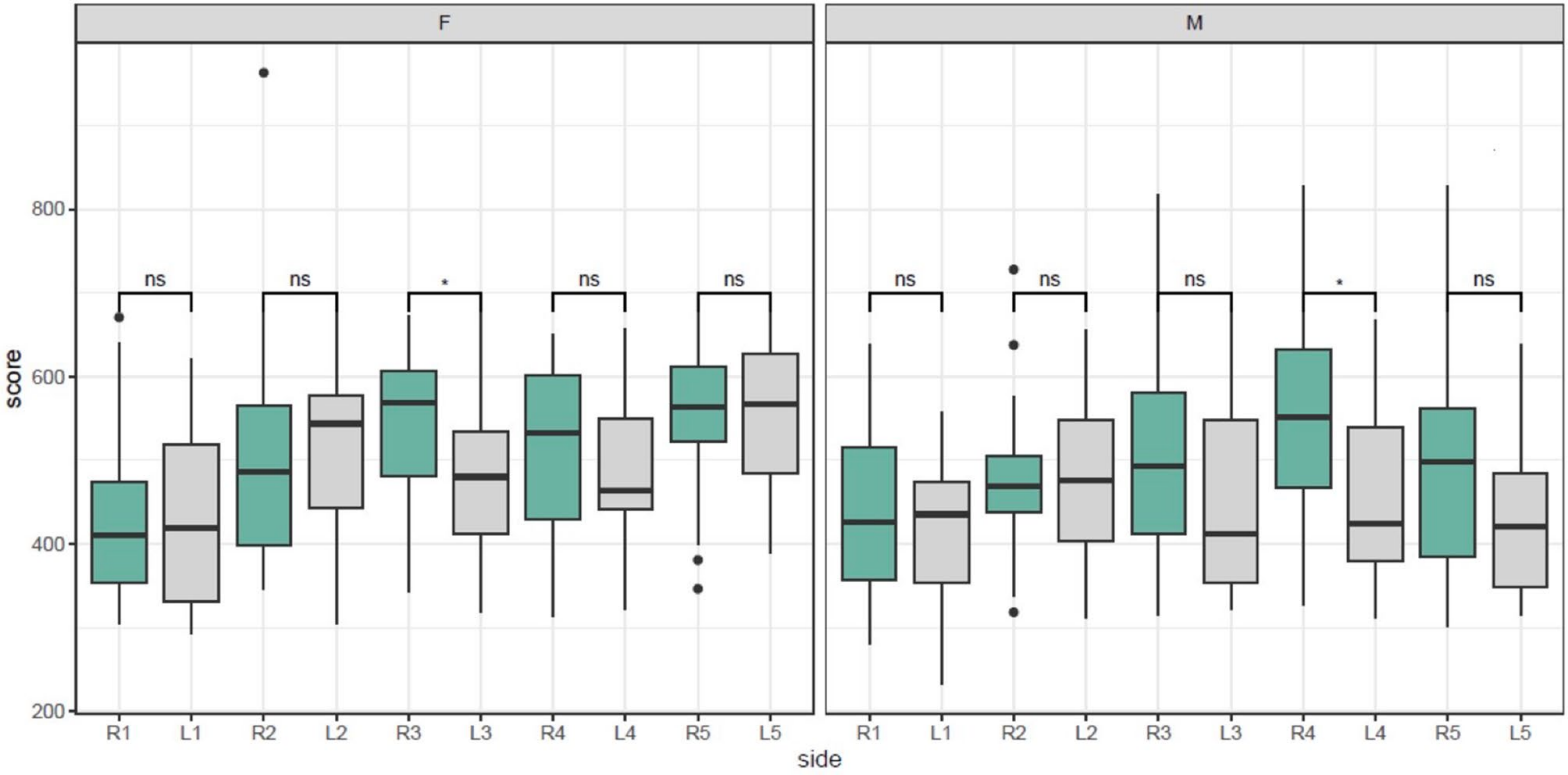
The average rigidity of the flexor tendons of the fingers of the hands by gender.

Interestingly, it was found that the flexor tendons of the ring finger were significantly more rigid than those of the fingers of the left hand (Graph 2) (Table 3). Moreover, when men and women were compared, it was found that the increased rigidity in the flexor tendons of the ring finger appeared only in the male population (Fig. 4).

**Table 3:** Comparison between flexor tendon of the right ring finger and the flexor tendons in each finger of the left hand.

Pairs (R = Right hand, L = Left hand, # = Finger number)	Mean difference (CI 95%)	t-test	p value
R4–L1	111.92 (57.37, 166.48)	4.16	0.000
R4–L2	43.35 (− 0.09, 86.80)	2.02	0.050
R4–L3	66.40 (3.80, 129.0)	2.15	0.038
R4–L4	62.86 (9.08, 116.63)	2.37	0.023

**Figure 5:**
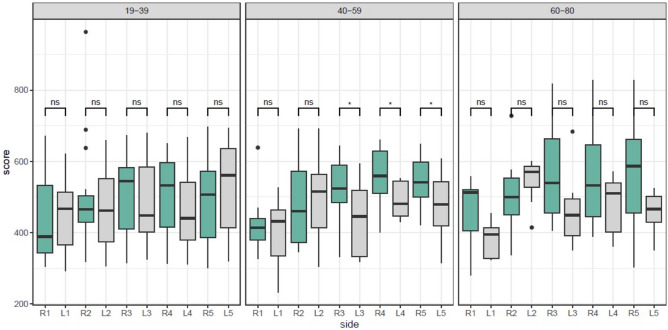
The average rigidity of the flexor tendons of the fingers of the hands by age groups.

Regarding the three different age groups, we found that there was no significant difference in flexor tendon rigidity in the fingers of the young age group (aged 19–39 years). However, in the middle-aged group (aged 40–59 years), there was an increased variation of flexor tendon rigidity, without a specific pattern, and this tendency decreased in the older aged group (aged 60–80 years) (Fig. 5).

## Discussion

In this observational study, we investigated the rigidity of hand flexor tendons through ultrasound, utilizing the SEG method, among healthy volunteers. Our objective was to establish baseline values for flexor tendon rigidity in the first pulley territory of the hand, creating a foundation for future comparisons with flexor tendon pathologies, such as trigger finger. Current knowledge suggests that in advanced pathological trigger fingers, tendons play a role in both the pathophysiological process and the narrowing of the first pulley^7,15^.

Studies from recent years have attempted to demonstrate the effectiveness of ultrasound through the sonoelastography method in revealing the degree of elasticity of tendons, particularly in the lower limbs^16,17^.

We identified that the average rigidity of the flexor tendons was higher in the right hand compared to the left hand. However, we observed no differences in tendon rigidity between the fingers of the same hand. Additionally, the flexor tendons of the thumbs exhibited distinct characteristics compared to those of the other fingers within the same hand; however, no significant difference was found between the flexors of the thumbs of the two hands. This finding may be explained by the fact that the thumb has one flexor tendon, whereas the other fingers have two.

Of note, in the male population, the flexor tendons in the ring finger of the dominant hand (which was the right hand for the majority—94%—of the subjects) were more rigid than the flexor tendons of the left-hand fingers, excluding the thumbs. This phenomenon could be attributed to the function of the dominant hand’s ring finger in daily activities, especially considering that men typically engage in more substantial gripping activities than women. However, it's essential to consider previous research of Radhakrishnan et al.^15^ indicating greater isometric strength in the index finger compared to the fourth finger^16^, warranting further investigation.

Moreover, when stratifying subjects into different age groups, the middle-aged group displayed a greater tendency for variation in flexor tendon rigidity, without a discernible pattern. This variability decreased in the younger and older age groups. This observation may be explained by the higher level of function and activity in middle-aged individuals, with long-term effects on tendon stiffness due to cumulative fingers activities. If this theory holds, we anticipate that the older age group would exhibit greater tendon rigidity, necessitating further investigation.

Several limitations were inherent in this study. The statistical test power was 80%, owing to the overall small sample size and the limited number of healthy volunteers in the older age group. Additionally, most participants had a dominant right hand, influencing the generalizability of the findings. Another limitation pertains to a single examiner conducting the ultrasound examination, without a comparison of results among different examiners.

Despite these limitations, this study holds clinical significance and has the potential to serve as a cornerstone for future investigations into flexor tendon pathologies. Furthermore, our results may guide surgeons in selecting the appropriate surgical intervention based on flexor tendon involvement, particularly in cases of advanced trigger finger.

Looking forward, these findings may equip hand surgeons with an additional non-invasive tool to aid in decision-making regarding the necessity of specific surgical interventions for patients with “trigger finger”. This could include determining whether a patient requires only a release of the first pulley or a combined surgery, such as resecting half of the superficial flexor tendon^17^ and releasing the first pulley, especially when presurgical ultrasound tests indicate a high level of rigidity.

## Data Availability

The datasets used and/or analyzed during the current study available from the corresponding author on reasonable request.
